# Multi-Sensor Fusion by CWT-PARAFAC-IPSO-SVM for Intelligent Mechanical Fault Diagnosis

**DOI:** 10.3390/s22103647

**Published:** 2022-05-10

**Authors:** Hanxin Chen, Shaoyi Li

**Affiliations:** 1 School of Artificial Intelligence, Nanchang Institute of Science and Technology, Nanchang 330108, China; lisy@ncpu.edu.cn; 2 School of Mechanical and Electrical Engineering, Wuhan Institute of Technology, Wuhan 430074, China

**Keywords:** fault diagnosis, PARAFAC, SVM, slurry pump, IPSO

## Abstract

A new method of multi-sensor signal analysis for fault diagnosis of centrifugal pump based on parallel factor analysis (PARAFAC) and support vector machine (SVM) is proposed. The single-channel vibration signal is analyzed by Continuous Wavelet Transform (CWT) to construct the time–frequency representation. The multiple time–frequency data are used to construct the three-dimension data matrix. The 3-level PARAFAC method is proposed to decompose the data matrix to obtain the six features, which are the time domain signal (mode 3) and frequency domain signal (mode 2) of each level within the three-level PARAFAC. The eighteen features from three direction vibration signals are used to test the data processing capability of the algorithm models by the comparison among the CWT-PARAFAC-IPSO-SVM, WPA-PSO-SVM, WPA-IPSO-SVM, and CWT-PARAFAC-PSO-SVM. The results show that the multi-channel three-level data decomposition with PARAFAC has better performance than WPT. The improved particle swarm optimization (IPSO) has a great improvement in the complexity of the optimization structure and running time compared to the conventional particle swarm optimization (PSO.) It verifies that the proposed CWT-PARAFAC-IPSO-SVM is the most optimal hybrid algorithm. Further, it is characteristic of its robust and reliable superiority to process the multiple sources of big data in continuous condition monitoring in the large-scale mechanical system.

## 1. Introduction

Fault diagnosis plays an important role in machine health management, which builds a bridge between data for machine monitoring and health status. Intelligent fault diagnosis uses artificial intelligence technology to make the process of fault diagnosis intelligent and automatic. Intelligent fault diagnosis is a promising topic in mechanical safety management, structural health monitoring, etc. [1].

A centrifugal pump, which is a very complex nonlinear system, plays a very critical role in industrial applications for continuous safe operation and production, especially during the industrial process of tranferring the oil sand. Muralidharan et al. [2] studied vibration-based continuous monitoring and analysis using a machine learning method based on the artificial neural network with fuzzy logic. The support vector machine algorithm is proposed for the continuous condition monitoring of centrifugal to extract the features from the vibration signals. Intelligent prognosis methods for remaining life in the condition-based maintenance of machinery are focused popularly nowadays. Khan et al. [3] developed a novel method to predict the remaining life of the industrial slurry pump, especially for solving the existing challenge in the ideal database, which is the data acquired from the start of running to the final failure of the machinery. A hybrid nonlinear autoregressive model was developed to utilize the prior obtained vibration signal from slurry pumps to generate degradation trends.

When the time domain analysis and frequency domain analysis can not meet the needs of extracting the characteristics of mechanical fault signals, the time–frequency analysis method combining time and frequency can effectively deal with non-stationary signals. It can master the information of the time domain and frequency domain at the same time, so as to find the characteristics of fault signals. Wavelet transform is a method used to obtain time domain detailed information by multi-scale analysis of fault signals through expansion, translation, and other operational functions. It removes the limitation of insufficient resolution in the time domain and frequency domain in the process of high-frequency information and low-frequency information by the short-time Fourier transform. Wavelet transform has a good localization analysis ability to play an important role in the fields of mechanical fault signal preprocessing, feature extraction, and fault diagnosis [4,5].

The time and frequency domain analysis is one of the conventional methods for the applications in mechanical fault diagnosis of the running status by the vibration signal analysis [6,7]. The vibration data collected from the machinery contains heavy noise and a large number of insensitive information. The insufficient capability in the existing method such as CWT about the characteristic signal recognition can not provide enough theoretical support for the reliably diagnosing the operating states of the mechanical devices [8,9].

PARAFAC technology has been widely used in signal processing and other fields. Multidimensional data decomposition combines the information of time, energy distribution, and space, and can extract the hidden information from the relationship between signals. Cheng et al. [10] proposed a novel method for blind source separation of the vibration signals to obtain the signal source number. Parallel factor analysis can provide an accurate assessment for the mixing array of the multiple source mixture of nonstationary conditions of the complicated mechanical system. Nguyen [11] proposed the method for the EEG analysis with PARAFAC and SVM to automatically classify the individuals by age and gender. PARAFAC has great advantages over the conventional methods such as Principle Component Analysis (PCA) in analyzing multi-dimensional data [12].

Pattern Recognition attracts broad attention as an important methodology in mechanical fault diagnosis. The SVM is one of the promising methods for intelligent fault diagnosis with a small number of data samples, which is used as pattern recognition classifiers [13]. Djeziri et al. proposed a temporal-based SVM approach for the detection and identification of pollutant gases in a gas mixture, which overcomes the challenges in the detection and control of gas pollution. Sing et.al used the tuned Support Vector Regression (SVR) model as the machine learning algorithm for prediction with a wireless sensor network [14]. An SVM model is proposed to be combined with the random forest model to solve the existing early detection task on fault detection and diagnosis of the malfunctioning of the heating, ventilating, and air conditioning system [15].

The swarm algorithms are widely applied in searching for an optimal solution. The big challenge in the conventional particle swarm optimization algorithm for application in miniatured devices and systems is the reduction of the energy consumption by the running hardware. The flexible and adaptive algorithm is proposed to reduce the structural complexity of hardware, obtain high-speed operation and reduce energy consumption [16]. The particle filter is used as the optimization method for the applications in intelligent detection systems and is normally combined with multidimensional data analysis [17,18]. Hu et al. proposed an indoor navigation algorithm using multi-dimensional Euclidean distance and an adaptive particle filter [19]. A particle swarm algorithm is used to optimize the weight values and threshold values of the wavelet neural network, which decreases the number of iterations to increase the speed and improves the convergence accuracy for good achievements [20]. The particle swarm optimization is proposed to be combined with SVM to identify the optimal feature subset for discriminating mental stress states, which improves the classification performance [21].

In this paper, the theory and algorithm about PARAFAC are studied. We studied the IPSO algorithm to optimize the parameters of SVM to establish the CWT-PARAFAC-IPSO-SVM model. The optimal comparisons among the classifier models are implemented. We utilize the advantages of PARAFAC, SVM, IPSO, WPA, and BP to develop hybrid methods, which are verified in the fault detection of slurry pumps.

### PARAFAC Algorithm

Parallel factorization is considered the multidimensional low-rank decomposition. The definition of parallel factorization was the first to be given for the data analysis in the field of psycho-experimental science. PARAFAC was used successfully in research areas such as chemical statistics, wireless communication, and blind source separation. The 3D data set $X\in C^{I\times J\times K}$ is decomposed into the sum of 3D matrices as shown in Equation (1) [22].

(1) $$X=\{(x_{i},y_{i}|i=1,2,\ldots ,N\},x_{i}\in R^{n},y_{i}\in (-1,1)$$

The low range decomposition process of the 3D matrix is identified by Equation (2). The rank of the 3D matrix is R and the cube is viewed by the 3D matrix X. The data matrix $S_{N_{e}\times N_{f}\times N_{g}}$ is the three-dimensional time-varying spectrum array $N_{e}$ obtained by wavelet transformation of vibration signal, $N_{f}$ and $N_{g}$ are the number of channels, frequency steps, and the number of data points, respectively.

**Figure 1:**
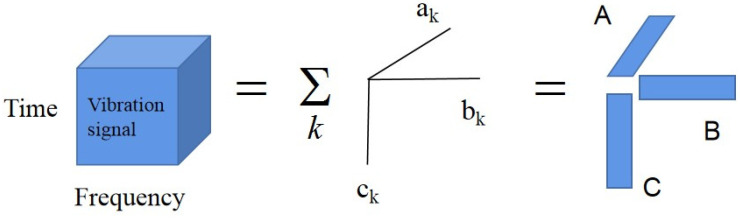
PARAFAC Decomposition Model $a_{ek}$, $b_{fk}$ and $c_{gk}$.

The main problem of this model is the characterization of the matrices A, B and C as shown in Figure 1. The elements are $a_{ek}$, $b_{fk}$ and $c_{gk}$. Each part k represents an atom. The associated vectors $a_{k}=\{a_{ek}\}$, $b_{k}=\{b_{fk}\}$, $c_{k}=\{c_{gk}\}$ are the spatial, spectral, and temporal signals of each atom. The decomposition in Equation (2) is achieved by solving $\underset{a_{ek}b_{fk}c_{gk}}{\min}{\hat{S}}_{efg}-\sum_{k=1}^{N_{k}}a_{ek}b_{fk}c_{gk}$. In PARAFAC, the $a_{k}(N_{e}\times 1)$ vector is regarded as the kth−dimension space vector, the $b_{k}(N_{f}\times 1)$ vector is regarded as the kth−component frequency, and the $c_{k}(N_{g}\times 1)$ vector is regarded as the kth−component of the time signals. The main benefit of this approach is that the spectral decomposition is unique and the best model is guaranteed to be obtained under the theory of the least square difference.

(2) $${\hat{S}}_{efg}=\sum_{k=1}^{N_{k}}a_{ek}b_{fk}c_{gk}$$

In this paper, the PARAFAC algorithm proposed is proposed as follows:

1. Time–frequency decomposition until convergence.
2. Find out the number of factors F
3. Initialize the load matrices B and C
4. *A* is estimated by the least square regression algorithm, that is, $A=XZ^{\prime }{\left(ZZ^{\prime }\right)}^{-1},\;Z=(b⊗c)$
5. Complete the same step for B and C
6. Continuously measure from step (3) before convergence.

## 2. Optimization of SVM parameters with Improved Particle Swarm Optimization (IPSO)

### 2.1. Principle of SVM

In the linearly divisible case, SVM is proposed from the optimal classification surface [23], assuming that the sample in the training set $X=\{(x_{i},y_{i}|i=1,2,\ldots ,N\},x_{i}\in R^{n},y_{i}\in (-1,1)$, Where $x_{i}$ is the indicator of input, $y_{i}$ is the indicator of output. The purpose of classification is to find a hyperplane that can entirely separate the two classes of samples for the two-class classification problem. The hyperplane is obtained by the nonlinear mapping: (ω⋅x)+b=0. It is vital not only to correctly separate the samples but also to increase the classification interval. Solving the optimal hyperplane classification is translated to solving the following problem of optimization:

(3) $$\left\{ \begin{array}{l} \min\Phi (\omega )=\frac{1}{2}{\left\|\omega \right\|}^{2}+C\sum_{i=1}^{N}\xi_{i}\\ y_{i}[\omega \cdot x_{i}+b]\geq 1-\xi_{i},\xi_{i}>0,i=1,2,\ldots ,N \end{array}\right.$$

where the parameter ω is the hyperplane’s weight vector. The parameter b is the bias. The parameter c is the penalty factor, which is one of the important factors affecting the classification of SVM performance. The parameter $\xi_{i}$ is the variable of relaxation. The Lagrangian function is introduced and the original problem of optimization is made into being the concept of pairs using the following Equation (4):

(4) $$\left\{ \begin{array}{l} \max Q(\alpha )=\sum_{i=1}^{N}\alpha_{i}-\frac{1}{2}\sum_{i,j=1}^{N}\alpha_{i}\alpha_{j}y_{i}y_{j}K(x_{i},x_{j})\\ \sum_{i=1}^{N}\alpha_{i}y_{i}=0,0\leq \alpha_{i}\leq C,i=1,2,\ldots ,N \end{array}\right.$$

The Lagrange multiplier is $\alpha_{i}$ and the kernel function is $K(x_{i},x_{j})$. The kernels of the functions commonly used in SVM are linear kernel function, polynomial kernel function, RBF kernel function, sigmoid activation functions, etc. We use the universal RBF kernel function, which has superiority as shown in the literature [15]. The expression of the function is:

(5) $$K(x_{i},x_{j})=\exp(-g{\left\|x_{i}-x_{j}\right\|}^{2})$$

Here g is a kernel factor that controls the Gaussian kernel’s range of action and is another parameter that affects the performance of the SVM classification. To obtain the decision function, the radial basis kernel function is used as:

(6) $$f(x)=\mathrm{sgn}(\sum_{i=1}^{N}\alpha_{i}y_{i}K(x_{i},x)+b)$$

### 2.2. Algorithm and Theory of IPSO

The PSO algorithm is similar to the genetic algorithm. It starts from the random solution and looks for the optimal solution through iteration. The quality of the solution through fitness is evaluated. The implementation of this algorithm is simpler and looks for the global optimal solution by following the current optimal value. This paper proposes an improved particle swarm optimization algorithm (IPSO) to optimize the super parameters of SVM. The algorithm adjusts the update mode of particles to simplify the particle swarm optimization algorithm. It has the advantage of accelerating the convergence speed in the later stage of particle swarm evolution and avoiding falling into local optimization to achieve good results.

IPSO is used to optimize the hyperparameters of the SVM. Based on the particle swarm optimization (PSO) algorithm shown in Equations (7) and (8), a new dynamic inertia weight and an optimized particle velocity and position update strategy are introduced to prevent the algorithm from dropping into the local optimum and boost the generalization efficiency of the SVM model.

(7) $$v_{id}^{k}=\omega_{i}v_{id}^{k-1}+c_{1}r_{1}(p_{bi}-x_{id}^{k-1})+c_{2}r_{2}(p_{g}-x_{id}^{k-1})$$

(8) $$x_{id}^{k}=x_{id}^{k-1}+v_{id}^{k}$$

Here, the parameter i=1,2,…,m and d=1,2,…,n, n is the dimensionality of the solution vector space, where m is the number of particles in the population, the parameters $c_{1}$ and $c_{2}$ are two positive constants, the parameter $r_{1}$ and $r_{2}$ are two independent random numbers between [0, 1], the parameter ω is the coefficient of momentum term, the parameter $p_{bi}$ denotes the optimal path experienced by the actual particle, the parameter $p_{g}$ denotes the position of the population’s ideal particle.

Following a boost in two aspects of the above general particle swarm algorithm [16], the IPSO algorithm is constructed.

1. An IPSO algorithm considers the effect of other population particles on the optimum search of the particles in the iteration. Each particle’s velocity is optimized according to the following three factors: the historical optimal value of the particles $p_{bi}$, optimal values of the particle $q_{b}$ within the neighborhood of the particles, and the global optimal value of the population $p_{g}$.

The distance between each particle and other particles is determined in the iteration. The distance between the current particle m and any particles n is specified as the parameter $l_{mn}$ and the maximum distance is the parameter $l_{\max}$. The ratio is calculated as $l_{mn}/l_{\max}$. According to the number of iterations, the threshold ξ varies and its description is

(9) $$\xi =\frac{0.3k+0.6k_{\max}}{k_{\max}}$$

where k determines the number of iterations. The maximum number of iterations is defined as the parameter $k_{\max}$. When the inequality ξ<0.9 and $l_{mn}/l_{\max}<\xi$ is satisfied, the particle n is found to be in the vicinity of $m^{th}$ particle. The introduction of the quality learning factor $c_{3}$ and the random number $r_{3}$, modifies the particle velocity according to the following equation.

(10) $$v_{id}^{k}=\omega_{i}v_{id}^{k-1}+c_{1}r_{1}(p_{bi}-x_{id}^{k-1})+c_{2}r_{2}(p_{g}-x_{id}^{k-1})+c_{3}r_{3}(q_{b}-x_{id}^{k-1})$$

If inequality ξ>0.9 or $l_{mn}/l_{\max}>\xi$ is satisfied, the speed of the particles is updated according to (7).

2. The standard PSO algorithm uses the parameter ω to decrease the phase length, which is determined by seeking linearly and gradually to converge the iterations to the extreme value point [7]. The drawback of this method is that the arithmetic pair is likely to collapse into the local optimum. To address these drawbacks, the parameter ω decrease as an S-shaped function and changes dynamically. The parameter ω is set to be a large value at the beginning of the optimization process to facilitate the global search and becomes smaller at the end of the search process to facilitate the local convergence. The representation of the weights in the IPSO algorithm is as follows:

(11) $$\omega =\frac{\omega_{\max}-\omega_{\min}}{1+\exp(2e\cdot t/t_{m}-e)}$$

**Figure 2:**
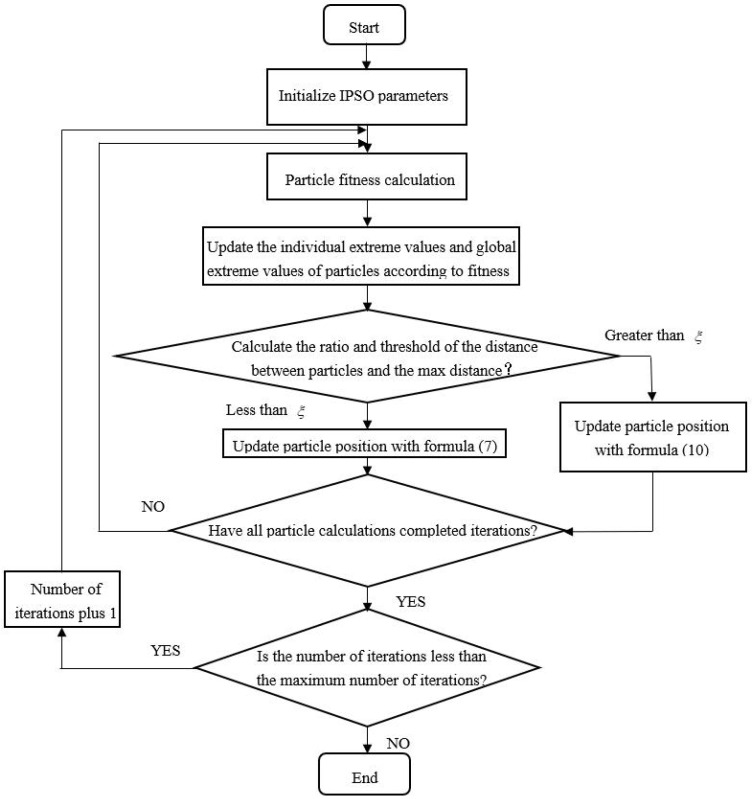
Procedure about IPSO-SVM algorithm.

The procedure of the IPSO algorithm is shown in Figure 2.

Step 1: Set the important IPSO parameters such as learning factor, the maximum number of iterations, population size, etc.

Step 2: Initialize the individual pole position of the particle $p_{bi}=(x_{i1},x_{i2},\ldots ,x_{in})$, the corresponding pole value $p_{bf}$, the position of the global pole $p_{g}=(x_{g1},x_{g2},\ldots ,x_{gn})$ , and the corresponding global pole value $p_{gf}$.

Step 3: Measure all $p_{i}$ values for particle fitness.

Step 4: the parameters $p_{bi}$, $p_{bf}$, $p_{g}$ and $p_{gf}$ are compared.

Step 5: Update the particles’ locations and keep them within their limits.

If $x_{ij}(k+1)>x_{\max}$, then $x_{ij}(k+1)=x_{\max}$;

If $x_{ij}(k+1)<x_{\min}$, then $x_{ij}(k+1)=x_{\min}$.

Otherwise, $x_{ij}(k+1)$ does not change.

Where the variables $x_{1}$ and $x_{2}$ are the maximum position and minimum position.

Step 6: Terminate the iteration if the number of iterations or the cutoff accuracy is satisfied; otherwise, return to Step 2.

## 3. The Experimental System of Slurry Pump

A slurry pump is characteristic of a very complex nonlinear mechanism. There are various failures of the slurry pump. The main impeller failures of the slurry pump are perforation damage (F2), outer edge wear (F3), and vane wear (F4), which are selected in the experiment to be compared with the normal impeller (F1).

**Figure 3:**
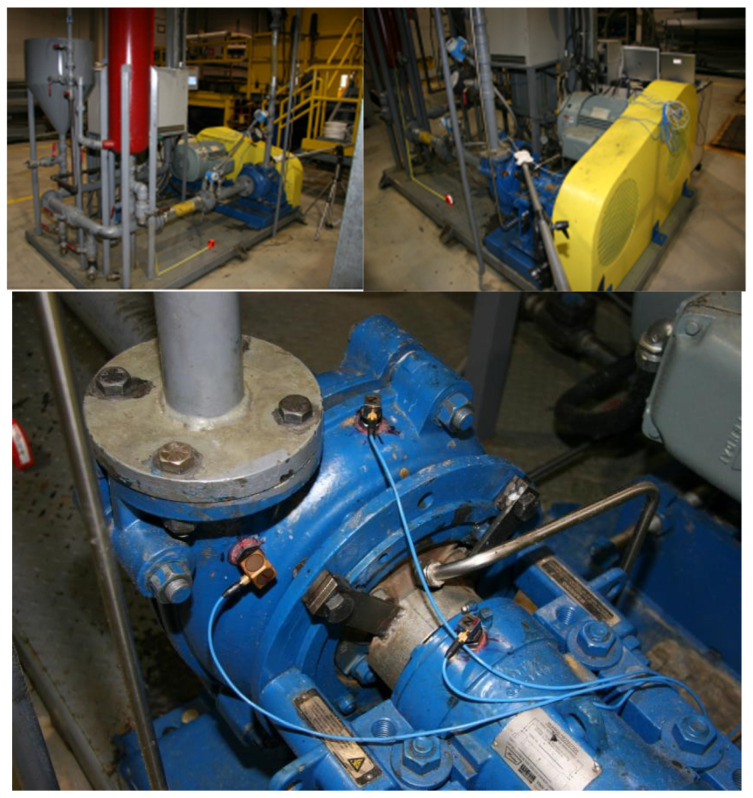
Experimental system of slurry pump with multiple channel sensors.

The multi-source dynamic condition monitoring of the mechanical system is established as shown in Figure 3. The whole vibration signal acquisition system is mainly composed of a signal analyzer and notebook computer storing data. The motor speeds are set to be 1200 rpm. In order to collect the vibration signals of the centrifugal pump in various states, it is necessary to install sensors at the key positions of the centrifugal pump and judge the vibration of the centrifugal pump through three acceleration sensors. As shown in Figure 3, one accelerometer with high sensitivity and low acquisition frequency is placed on the top of the pump, and the other two accelerometers with low sensitivity and high acquisition frequency are placed on the outlet of the pump and on the top of the bearing, respectively.

The centrifugal pump under different working conditions is simulated by replacing different impellers and changing the rotational speed. The vibration signals are collected by the accelerometers. The steps are as follows: (1) The normal centrifugal pump was used in the experiment and operated stably for a period of time. We carefully check all parts of the centrifugal pump to ensure that the centrifugal pump is in good condition and replace the impeller of the centrifugal pump with the normal impeller F1. After the centrifugal pump is idling and stable, we open the inlet pipe valve to introduce mud and then adjust the impeller speed to 1200 rpm according to the transmission ratio. When the outlet pressure of the pump is higher than the operating pressure, we gradually open the outlet valve. The centrifugal pump operates stably and the experimental data are collected by the signal acquisition system. The data acquisition time of each group is 20 s and the acquisition frequency is 9KHz. (2) The faulty impeller F2 is selected to replace the impeller in the original centrifugal pump and other parts remain unchanged. The other running conditions remain unchanged, and we collect the experimental data of fault impeller F2 according to the method of step (1). (3) The faulty impeller F3 is selected to replace the impeller in the original centrifugal pump and other parts remain unchanged. The other running system conditions remain unchanged. We collect the experimental data of fault impeller F3 according to the method of step (1). (4) The faulty impeller F4 is selected to replace the impeller in the original centrifugal pump and other parts remain unchanged. The other running conditions remain unchanged. We collect the experimental data of fault impeller F4 according to the method of step (1). (5) After the above experimental procedure, we stop the machine according to the standard process. We store the experimental data to prepare for the subsequent vibration signal analysis.

## 4. CWT-PARAFAC-IPSO-SVM for Fault Diagnosis

### 4.1. Multi-Channel Vibration Signal Analysis with PARAFAC

**Figure 4:**
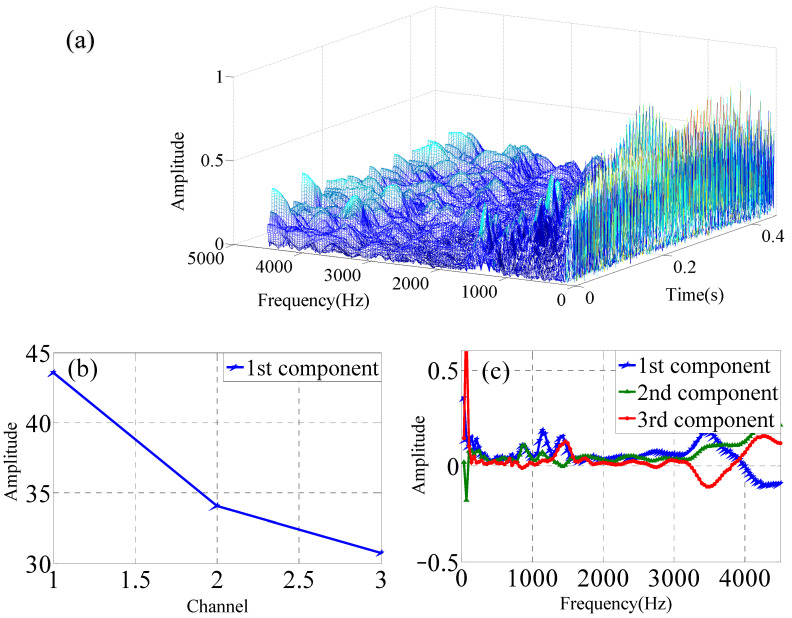

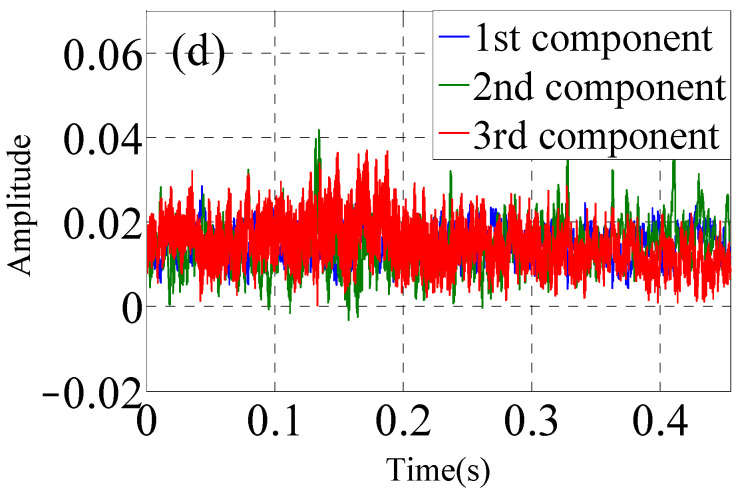
PARAFAC for one data from one channel of single sensor under F1 condition, (**a**) CWT of single-channel data, (**b**) mode 1, (**c**) mode 2, (**d**) mode 3.

Single-channel vibration signals are collected at one *X*-axis measuring point of one accelerometer. The single-channel data are used to construct the three-dimensional data matrix by three experimental vibration data that are collected by one accelerometer. The single-channel sensor data are analyzed by Continous Wavelet Transform (CWT) as shown in Figure 4a. The time–frequency domain data matrix is analyzed by PARAFAC decomposition to obtain the three modes, which is shown in Figure 4b,c for the normal impeller (F1).

**Figure 5:**
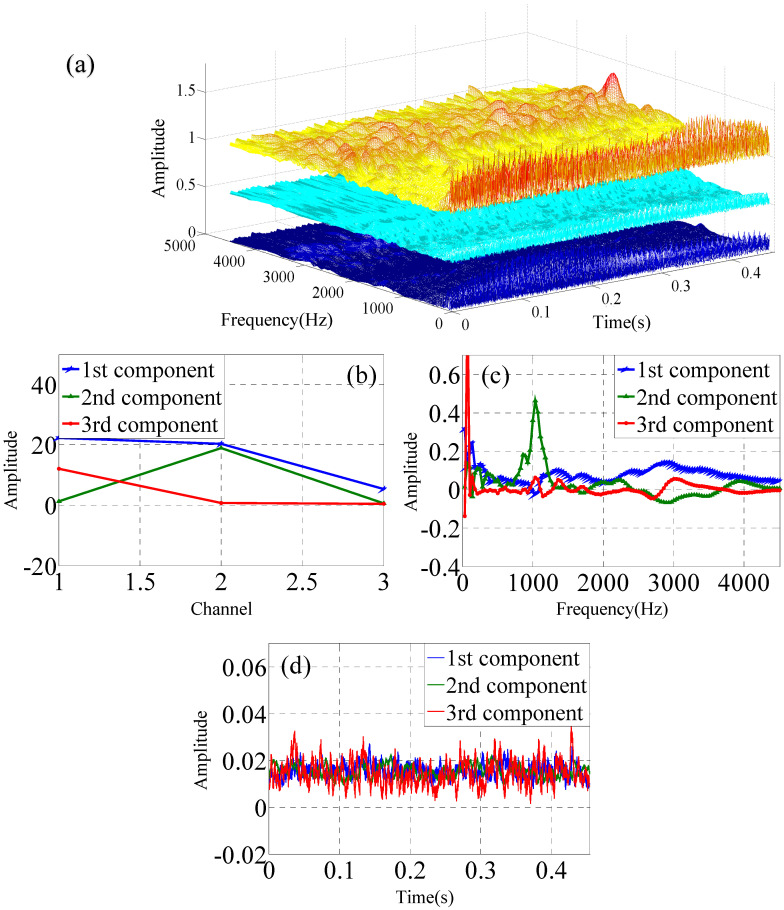
FARAFAC for time–frequency–space data matrix from *x*-*y*-*z* axes channel of one sensor under F1 condition, (**a**) Time–frequency–space with CWT of three-channel data of one accelerometer, (**b**) mode 1, (**c**) mode 2, (**d**) mode 3.

**Figure 6:**
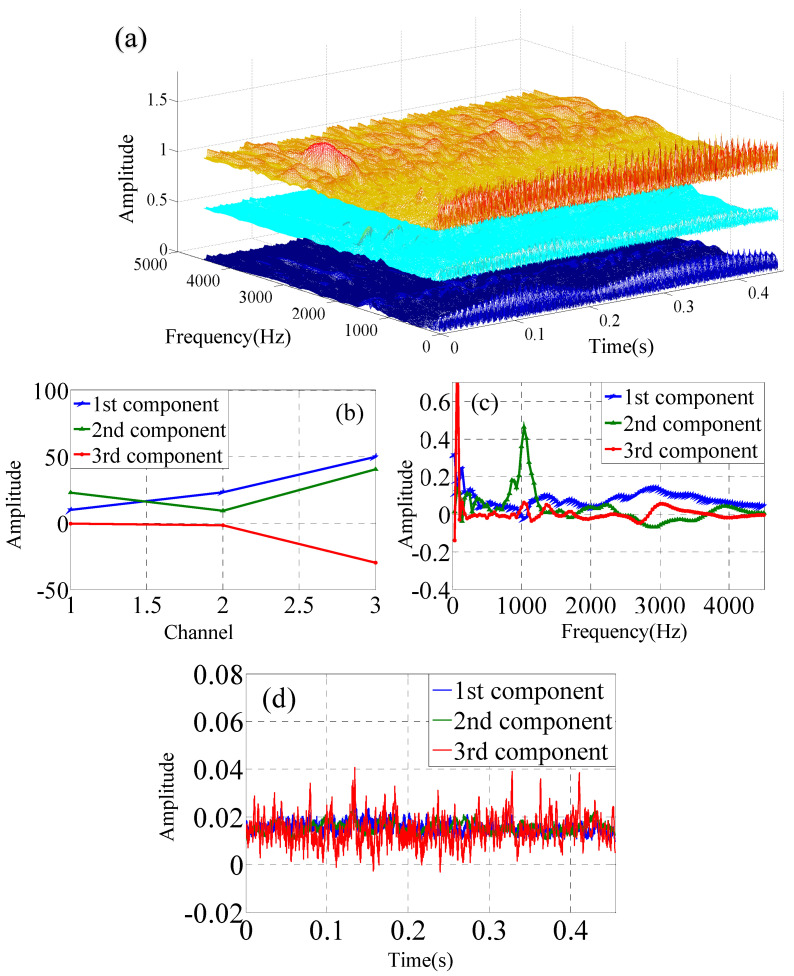
PARAFAC for time–frequency–space data matrix from three sensors under F1 condition. (**a**) Time–frequency–space with CWT of three-channel data, (**b**) mode 1, (**b**,**c**) mode 2, (**d**) mode 3.

Multi-channel vibration signals include two categories. The one consists of the *x*-*y*-*z* axis at one measurement point of one accelerometer in the operating condition of the slurry pump, which is shown in Figure 5. Another one consists of the three *x*-axis measurement points of the three accelerometers in the operating condition of the slurry pump, which is shown in Figure 6. The data from one channel is transformed by CWT. The three-channel data are used to construct the three-dimensional time–frequency–space data matrix that is collected by accelerometers simultaneously. The three-dimensional data matrix is analyzed by the PARAFAC decomposition to obtain the three modes.

The channel loading mode 1, frequency loading mode 2, and time loading mode 3 are obtained after PARAFAC. Mode 2 and mode 3 accurately describe the normal or fault state of the devices by the empirical tests. The PARAFAC function model is used to evaluate the mapping relationship between the operating conditions of the slurry pumps and the corresponding mode 2 and mode 3. The mode 2 and mode 3 components of the three-level loading factors are extracted from the vibration signals under four conditions to construct a feature vector with six parameters.

**Figure 7:**
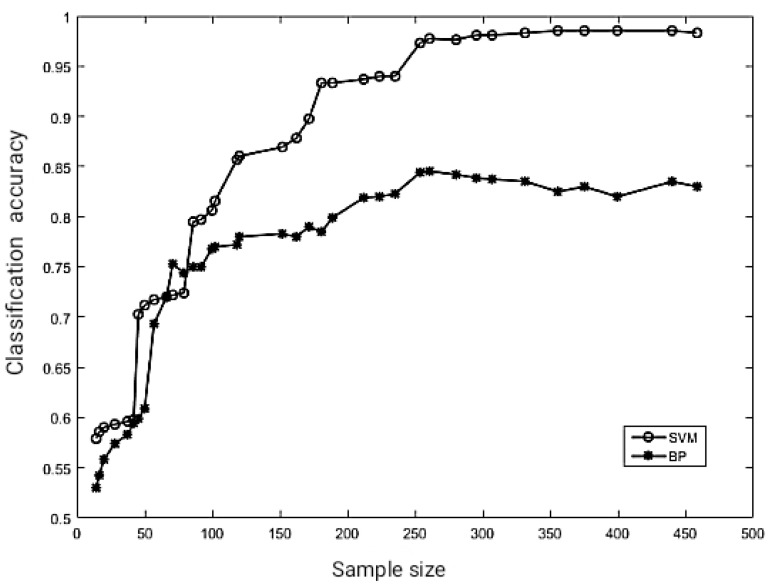
The relationship between classifier accuracy and the number of training samples.

The feature vectors with six parameters are used as input values to SVM and BP. Two classes of slurry pumps under the two fault states are chosen at random. It is demonstrated that the SVM classifier is much better than the BP neural network based on the classification success rates as shown in Figure 7. When the training set samples are greater than 120, the SVM classifier’s classification accuracy reaches more than 85%. When the BP neural network classifier’s training set samples are about 250, the classification accuracy is similar to 85%. The classification accuracy of the BP neural network has not improved with the increase in training samples. It shows that SVMs are more suited for classification with small samples.

### 4.2. Energy Feature Selection by WPD

The following two feature extraction was used to obtain the input vectors of the support vector machine classifier to test the classification output of the SVM classifier with various fault feature inputs, which is the energy after decomposition of the wavelet packet and the features extracted by PARAFAC decomposition from the multi-source signal. In Table 1, the SVM output and the corresponding slurry pump state are shown.

The 9000 data points of the vibration signals along the *X*-axis direction are collected at one measurement point under each running condition of the slurry pump. The noise of the raw vibration signal is reduced by wavelet packets. The energy of the original data that is analyzed by the wavelet packet decomposition is used as the input vector of the classifier. Because the energy of each frequency band in WPD under the four-state modes of the slurry pump is different, WPD is used to project the raw signal on the different frequency bands. The experimental signal energy is separated into the different frequency bands after the decomposition of the wavelet packet, which are used as the vectors of the fault functions. The three-level wavelet packet decomposition on the vibration signal is performed by using the wavelet function that is Daubechies6(D6). The coefficients of WPD are obtained from the three levels, which have eight frequency bands. The decomposition coefficients of the wavelet packet are reconstructed to obtain the eight new time-series signals within the eight frequency bands of the three levels, which are distributed in the sequence from high-frequency components to low-frequency components, which is denoted as $c_{0}(t),c_{1}(t),\ldots ,c_{7}(t)$. The total energy $E_{i}$ of each component is calculated as:

(12) $$E_{i}=\int_{-\infty }^{+\infty }{\left|c_{i}(t)\right|}^{2}dt,i=0,1,\ldots ,7$$

The vector T function with energy as an element is defined as:

(13) $$T=[E_{0},E_{1},E_{2},E_{3},E_{4},E_{5},E_{6},E_{7}]$$

The characteristic vectors are normalized in such a way by the following equations.

(14) $$E={\left(\sum_{i=1}^{8}{\left|E_{i}\right|}^{2}\right)}^{\frac{1}{2}}$$

(15) $$T^{\prime }=[E_{0}/E,E_{1}/E,\ldots ,E_{7}/E]$$

There are 60 groups of single-channel vibration data for each operating condition, which is collected by one accelerometer. The energy characteristic parameters of the wavelet packet are obtained by Equation (15). It is used as the input feature vector of the SVM model to verify the effectiveness and accuracy of the wavelet packet energy feature in fault diagnosis of centrifugal pump.

### 4.3. Parameter Optimization of SVM without IPSO by WPA Energy

In this paper, the RBF kernel function is selected as the classification kernel function. The initial values of the penalty factor and kernel function width are selected according to experience. In order to understand the effects of the penalty function (c) and the radial kernel function (g) on the recognition accuracy of the classifiers, twenty sets of WPA energy features were chosen as training samples acquired from the healthy condition of the slurry pump (F1) and the faulty condition of the slurry pump with the perforated impeller (F2), which means forty sets of samples in total. Each set of WPA energy features consists of seven parameters as shown in Equation (15). The value of the radial kernel function of the RBF (g) is two. The values of the penalty function (c) are selected to be 0.1, 2, 10, 50, and 100 to assess the performance of the SVM classifier without PSO, which is shown in Table 2.

Table 2 demonstrates that when the value of the penalty function (c) equals two, the accuracy of the classification vector is the highest. There is not much variation in the overall training time for different values of the penalty function (c). The number of support vectors needs to be increased as the c value becomes larger. Obviously, the option of the penalty function c has a major influence on the classification correct rate of SVM outputs.

It is necessary to assess the influence of radial kernel function (g) on the recognition accuracy of the SVM, twenty sets of energy features were chosen as training samples acquired from the healthy condition of the slurry pump (F1) and the faulty condition of the slurry pump with the perforated impeller (F2), which means forty sets of samples in total. Based on the above results in Table 2, the value of the penalty function (c) is set to two. The values of the radial kernel function of the RBF (g) are set to be 0.01, 0.1, 1, 10, and 20 for training the SVM classifiers without PSO. Table 3 shows that the correction rate is highest when the value of parameter c is 1.

Based on results in Table 2 and Table 3, penalty factor c equals two and ten and kernel function width g equals one and ten, which is much better for the running states classification of the slurry pump. The feature extraction capability with WPA energy as defined in Equation (15) needs to be assessed in combination with the SVM multi-classifier without PSO. There are four types of operating conditions, which are F1, F2, F3, and F4. Sixty sets of single-channel vibration signals are collected from the slurry pump, which means the total number of the vibration signal sets is 240. The size of the training feature vector samples is 120. The size of the testing feature vector sample is 120. The optimal values of RBF kernel function (g) and penalty function (c) are used to test the multiple SVM without PSO optimization. As shown in Table 4, the correction rates of the SVM classifiers based on WPA energy features of the single-source vibration signals are equal to or less than 80%. Table 2, Table 3 and Table 4 show that the optimal values of penalty factor c and kernel function width g of SVM classifiers are two and one for the operating conditions identification of the slurry pump.

### 4.4. Optimization of SVM Multi-Classifier without PSO by PARAFAC

As discussed in Section 4.1, the comparison in the feature extraction of the single-channel vibration signal analysis by PARAFAC between SVM and BP is presented. Mode 2 and mode 3 of PARAFAC have a relationship with the running states of the slurry pump. It is possible to determine the mapping relationship between modes 2–3 and operating conditions of the slurry pump with PARAFAC decomposition of the single-channel vibration signals.

The six features were extracted based on mode 2 and mode 3 of the three-level components of the PARAFAC analysis of the vibration signals collected from the slurry pump under the four operating conditions. In order to verify the classification accuracy of the extracted fault characteristics in the SVM classifier without PSO, 60 sets of the vibration signals were tested for each of the four conditions of the slurry pump and 240 sets of data in total. The number of the training samples is 120 and the testing samples are 120. The multi-classification model of the SVM is constructed by training the samples. The parameters that c=2 ,10 and g=1, 10 are used to classify the operating conditions of the slurry pump. Table 5 shows the correction rate of the classification by SVM without PSO by using the PARAFAC features. The classification accuracy is 83% for the training sets and 85% for the testing sets, which needs to be improved the classification accuracy substantially.

### 4.5. SVM Optimization with IPSO

The above classification results for the different features demonstrate the classification accuracy of the fault diagnosis model does not meet the application-level requirements by setting the model hyperparameters of SVM empirically in condition monitoring of the slurry pump. The IPSO algorithm is proposed to be used to optimize the SVM’s kernel function to make the classifier model optimal. The parameters are set as follows: $c_{1}=1.5$, $c_{2}=2$, $c_{3}=1,5$ and control coefficient e=8 are the significant criteria of the IPSO algorithm.

**Figure 8:**
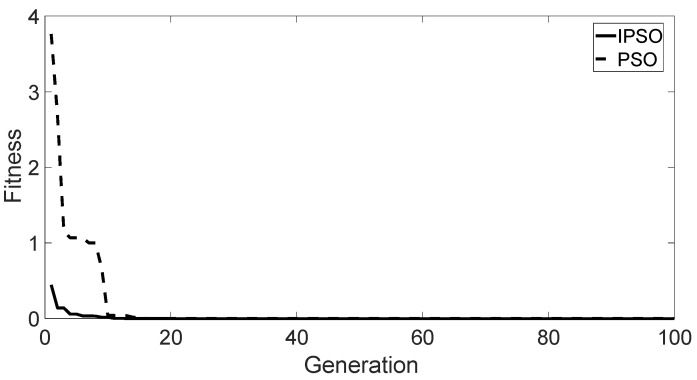
Fitness of Ackley function optimization.

**Figure 9:**
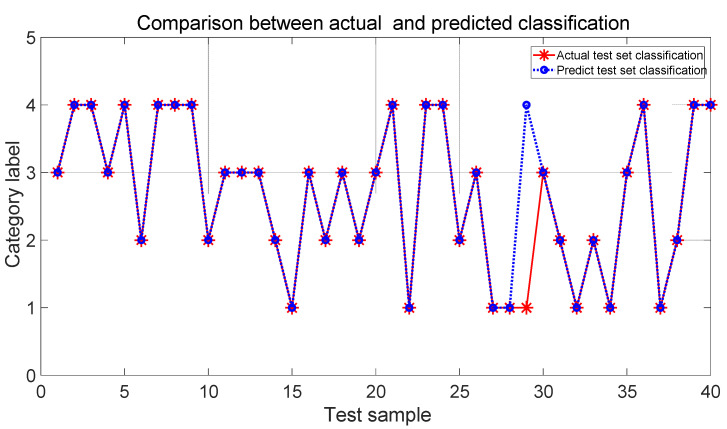
Classification observation based on SVM model.

The typical test function is the Ackley function, which is used to evaluate the reasonableness and effectiveness of the IPSO algorithm. The convergence curve of the optimization search is shown in Figure 8. Ultimately, the IPSO algorithm reaches the global optimum in about 10 cycles, which has a fast convergence with reasonably stable and robust results. For the SVM classifier, the IPSO algorithm was applied to optimize the SVM. The comparison of the classification success rates between the actual test set and the prediction test set is shown in Figure 9.

**Figure 10:**
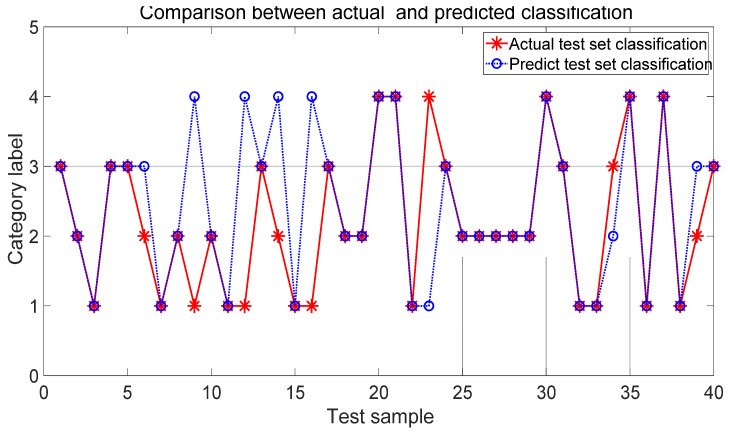
Classification with BP neural network.

The efficacy of the IPSO-SVM model was assessed to demonstrate the advantages by comparison with the BP network. The configuration of the BP neural network was 6-5-4. The maximum number of iterations was set to be 100. The learning rate was 0.01. The training goal was 0.001. There are 120 data sets, which are randomly chosen as the training samples. There are 40 testing data sets. Figure 10 shows the comparison of the classification between the actual and predicted classification by the BP network.

Based on the classification comparison in Figure 9 and Figure 10, it is shown that the developed IPSO-SVM classification model is much better than that of the BP neural network in classification rates, which meets the requirements of the application level. The model is stable and effective to improve the accuracy of recognizing the fault conditions.

## 5. PARAFAC-SVM with IPSO Optimization for Multi-Channel Data Analysis

In Table 4, the correction rate of four operating conditions identification is 75% and 79.2% by using the WPA energy as feature values and SVM without PSO as multiple classifiers. The optimal value for the penalty function (c) and RBF kernel function width (g) equal to two and one. In Table 5, the correction rate of four operating conditions identification is 83% and 85% by using the PARAFAC loading factors as feature values and SVM without PSO as multiple classifiers. The optimal value for the penalty function (c) and RBF kernel function width (g) equal to two and one. It is concluded that the penalty function and RBF kernel function width are two and one, which are used as the optimal parameter values for the following IPSO-SVM classifiers. Table 4 and Table 5 show PARAFAC has advantages over WPT for feature extraction when they are combined with SVM to construct the classifiers for fault diagnosis.

The above discussions about WPT-SVM, PARAFAC-SVM, PARAFAC-BP show that PARAFAC and SVM are much better performance in capability in classifications of the fault conditions than WPT and BP, which are used to extract the feature from vibration signal and recognize the conditions. The reason is that PARAFAC is characteristic of the multi-dimensional signal analysis from multiple source measurement points. PARAFAC is good at reducing the bad inter-inference between the multiple signal channels to obtain the intrinsic information, which represents the intrinsic physical mechanism.

In order to verify the capability in the classification accuracy with the WPA-IPSO-SVM classifier, 60 sets of the vibration signals were tested for each of the four conditions of the slurry pump. There are 240 sets of data in total. The number of the training samples is 120 and the testing samples are 120. The feature extraction with WPA energy as defined in Equation (15) is combined with the PSO-SVM multi-classifier to construct the WPA-PSO-SVM classifier and WPA-IPSO-SVM classifier.

The PARAFAC method used as feature extraction consists of single-channel data analysis and multi-channel data analysis, which is described in Section 4.1. PARAFAC is combined with PSO-SVM multi-classifier to construct the CWT-PARAFAC-PSO-SVM classifier and CWT-PARAFAC-IPSO-SVM classifier. The six features were extracted based on mode 2 and mode 3 of the three-level components of the PARAFAC analysis of the vibration signals collected from the slurry pump under the four operating conditions.

In order to improve the performance and capability of WPT-SVM and PARAFAC-SVM, IPSO is proposed to optimize SVM. As shown in Table 6, the correction rates of classifications by WPA-PSO-SVM and WPA-IPSO-SVM are 90%, 89.2%, and 92.5%, 93.2% for the training set and testing set for the single-channel vibration data analysis, which shows that PSO has the capability in improving the performance of SVM. IPSO has a great improvement over PSO.

In Table 6, the correction rates of classifications by CWT-PARAFAC-PSO-SVM and CWT-PARAFAC-IPSO-SVM are 94.2%, 92.5%, and 95.8%, 96.7% for the training set and testing set for the single-channel vibration data analysis, which shows IPSO has great improvement in optimization of SVM than PSO.

As described in Section 4.1, the multi-channel experimental vibration data are analyzed by FAPARAC. Table 7 shows the correction rate of the classifiers that is CWT-PARAFAC-PSO-SVM and CWT-PARAFAC-IPSO-SVM. The correction rates of CWT-PARAFAC-PSO-SVM and CWT-PARAFAC-IPSO-SVM are 96.7%, 95.8%, 100%, and 99.2%. By the comparison between Table 6 and Table 7, it is verified that the correction rates in Table 7 are much better than that in Table 6. The PARAFAC has an overwhelming capability for handling multi-dimensional data. Multiple-channel experimental vibration data contains more intrinsic information related to the operating conditions than single-channel vibration data. Particularly, PARAFAC can eliminate the information interference and information redundancy among various data channels and delete the insensitive system information to the faulty components of the nonstationary mechanical operation conditions.

Based on Table 6 and Table 7, it is verified that PARAFAC has a great advantage in analyzing source data, which can be used to improve the correction rates of operating condition identification. IPSO can improve the optimization of SVM parameters. The CWT-PARAFAC-IPSO-SVM fully utilizes the advantages of PARAFAC and IPSO. It is proven that CWT-PARAFAC-IPSO-SVM has strong merit for multi-channel big data analysis with around a 100% correction rate of operating condition identification in the nonstationary mechanical condition monitoring.

## 6. Conclusions

This paper proposes a novel method for feature extraction based on PARAFAC, which has outstanding performance in the multi-source vibration signal decomposition. The PSO is improved to construct the IPSO to optimize the SVM to develop the CWT-PARAFAC-IPSO-SVM for the intelligent fault diagnosis of the slurry pump. The hybrid method based on optimized PARAFAC-WPA_SVM by IPSO is proposed for fault diagnosis, which increases the correction rates of fault diagnosis up to 100%. It is shown that the proposed method based on the IPSO, WPS, PARAFAC, and SVM effectively increases the diagnostic accuracy and reduces the diagnosis time with no noticeable increase in complexity, which is compared with the conventional time domain and frequency domain feature extraction methods. In future work, we aim to study the effects of the key functions on the correction rates of fault diagnosis to find the optimal parameters and models.

## Figures and Tables

**Table 1:** SVM output for each state of slurry pump.

Running State	Normal	Perforation Damage	Outer Edge Wear	Blade Wear
SVM classifier output	[1]	[2]	[3]	[4]

**Table 2:** Effect of penalty functions (c) on SVM bi-classifier accuracy.

c	SVM Number	Time(s)	Correction Rate (%)
0.1	16	0.2316	67.5
2	19	0.2056	75
10	21	0.1865	65
50	20	0.1762	65
100	21	0.1723	62.5

**Table 3:** Effect of penalty function (g) on SVM bi-classifier accuracy.

g	SVM Number	Time(s)	Correction Rate (%)
0.01	17	0.2033	67.5
0.1	18	0.1801	70
1	20	0.1766	80
10	20	0.1923	70.5
20	20	0.2205	62.5

**Table 4:** Correction rate by SVM multi-classifier with WPA energy.

c	g	Training Time(s)	Training Data (%)	Testing Data (%)
2	1	1.8975	75	79.2
	10	1.557	71.67	74.2
10	1	1.857	70.8	69.2
	10	1.7895	69.2	73.3

**Table 5:** Correction rate by SVM multi-classifier with PARAFAC.

c	g	Training Time(s)	Training Data (%)	Testing Data (%)
2	1	1.3875	83	85
	10	1.3695	81.67	82.5
10	1	1.5675	80	80
	10	1.5315	78.3	77.5

**Table 6:** Correction rate of four classifiers with single-channel data analysis.

Classifier	Training Data (%)	Testing Data (%)	Time(s)
WPT-PSO-SVM	90	89.2	4.783
WPT-IPSO-SVM	92.5	93.3	5.729
CWT-PARAFAC-PSO-SVM	94.2	92.5	13.167
CWT-PARAFAC-IPSO-SVM	95.8	96.7	8.931

**Table 7:** Correction rate of two classifiers with multi-channel data analysis.

Classifier	Training Data (%)	Testing Data (%)	Time(s)
CWT-PARAFAC-PSO-SVM	96.7	95.8	12.853
CWT-PARAFAC-IPSO-SVM	100	99.2	9.462

## Data Availability

Not applicable.

## References

[1] Lei Y., Yang B., Jiang X., Jia F., Li N., Nandi A.K. (2020). Applications of machine learning to machine fault diagnosis: A review and roadmap. Mech. Syst. Signal Process..

[2] Muralidharan V., Sugumaran V., Sakthivel N.R. (2011). Wavelet decomposition and support vector machine for fault diagnosis of monoblock centrifugal pump. Int. J. Data Anal. Tech. Strateg..

[3] Khan M.M., Tse P.W., Trappey A.J.C. (2021). Development of a Novel Methodology for Remaining Useful Life Prediction of Industrial Slurry Pumps in the Absence of Run to Failure Data. Sensors.

[4] Li Q., Zhou Y.U., Tang G., Xin C., Zhang T. (2021). Early weak fault diagnosis of rolling bearing based on multilayer reconstruction filter. Shock. Vib..

[5] Cheng J., Yang Y., Li X., Cheng J. (2021). Adaptive periodic mode decomposition and its application in rolling bearing fault diagnosis. Mech. Syst. Signal Process..

[6] Hanxin C. (2021). Intelligent Model-based Integrity Assessment of Nonstationary Mechanical System. J. Web Eng..

[7] Chen H., Huang W., Huang J., Cao C., Yang L., He Y., Zeng L. (2019). Multi-fault condition monitoring of slurry pump with principle component analysis and sequential hypothesis test. Int. J. Patt. Recogn. Artif. Intell..

[8] Lin S.L. (2021). The Application of Machine Learning ICA-VMD in an Intelligent Diagnosis System in a Low SNR Environment. Sensors.

[9] Liu T., Xu H., Ragulskis M., Cao M., Ostachowicz W. (2020). A Data-Driven Damage Identification Framework Based on Transmissibility Function Datasets and One-Dimensional Convolutional Neural Networks: Verification on a Structural Health Monitoring Benchmark Structure. Sensors.

[10] Cheng Y., Li Z., Jin Y., Zhang X. (2017). Blind source separation of multi mixed vibration signal based on parallel factor analysis. Progn. Syst. Health Manag..

[11] Nguyen P., Tran D., Vo T., Huang X., Ma W., Phung D. (2013). EEG-Based Age and Gender Recognition Using Tensor Decomposition and Speech Features. Proceedings of the International Conference on Neural Information.

[12] Mingming L., Menglong L., Hanxin C., Yao K. Fault diagnosis method of centrifugal pump based on PARAFAC-SVM. Proceedings of the International Workshop on Automation, Control, and Communication Engineering.

[13] Zhang T., Chen J., Li F., Zhang K., Lv H., He S., Xu E. (2022). Intelligent fault diagnosis of machines with small and imbalanced data: A state-of-the-art review and possible extensions. ISA Trans..

[14] Djeziri M.A., Djedidi O., Morati N., Seguin J.-L., Bendahan M., Contaret T. (2021). A temporal-based SVM approach for the detection and identification of pollutant gases in a gas mixture. Appl. Intell..

[15] Tun W., Wong J.K.W., Ling S.H. (2021). Hybrid Random Forest and Support Vector Machine Modeling for HVAC Fault Detection and Diagnosis. Sensors.

[16] Długosz Z., Rajewski M., Długosz R., Talaśka T. (2021). A Novel, Low Computational Complexity, Parallel Swarm Algorithm for Application in Low-Energy Devices. Sensors.

[17] Mohamed A., Mohamed R., Elkomy O., Abouhawwash M. (2022). Recent metaheuristic algorithms with genetic operators for high-dimensional knapsack instances: A comparative study. Comput. Ind. Eng..

[18] Bacanin N., Zivkovic M., Bezdan T., Venkatachalam K., Abouhawwash M. (2022). Modified firefly aogorithm for workflow scheduling in cloud-edge environment. Neural Comput. Appl..

[19] Hu Y., Peng A., Tang B., Xu H. (2021). An Indoor Navigation Algorithm Using Multi-Dimensional Euclidean Distance and an Adaptive Particle Filter. Sensors.

[20] Chen H., Fang L., Liang Fan D., Huang W., Huang J., Cao C., Yang L., He Y., Zeng L. (2019). Particle swarm optimization algorithm with mutation operator for particle filter noise reduction in mechanical fault diagnosis. Int. J. Patt. Recogn. Artif. Intell..

[21] Hag A., Handayani D., Altalhi M., Pillai T., Mantoro T., Kit M.H., Al-Shargie F. (2021). Enhancing EEG-Based Mental Stress State Recognition Using an Improved Hybrid Feature Selection Algorithm. Sensors.

[22] Vapnik V.N. (1995). The Nature of Statistical Learning Theory.

[23] Wang J., Zhang W. (2015). Support Vector Machine Modeling and Intelligent Optimization.

